# A review on the potential of filamentous fungi for microbial self-healing of concrete

**DOI:** 10.1186/s40694-021-00122-7

**Published:** 2021-11-18

**Authors:** Aurélie Van Wylick, Antonielle Vieira Monclaro, Elise Elsacker, Simon Vandelook, Hubert Rahier, Lars De Laet, David Cannella, Eveline Peeters

**Affiliations:** 1 Research Group of Architectural Engineering, Department of Architectural Engineering, Vrije Universiteit Brussel, Pleinlaan 2, B-1050 Brussels, Belgium; 2 Research Group of Microbiology, Department of Bioengineering Sciences, Vrije Universiteit Brussel, Pleinlaan 2, B-1050 Brussels, Belgium; 3 PhotoBioCatalysis Unit–BTL–Ecole interfacultaire de Bioingénieurs (EIB), Université Libre de Bruxelles, Avenue F.D. Roosevelt 50, B-1050 Brussels, Belgium; 4 Research Group of Physical Chemistry and Polymer Science, Department of Materials and Chemistry, Vrije Universiteit Brussel, Pleinlaan 2, B-1050 Brussels, Belgium; 5 Newcastle University, Hub for Biotechnology in the Built Environment, Devonshire Building, Newcastle upon Tyne, NE1 7RU UK; 6 Present Address: Center for Microbial Ecology and Technology (CMET), Department of Biotechnology Faculty of Bioscience Engineering, Ghent University, Coupure Links 653, B-9000 Ghent, Belgium; 7 Present Address: Center for Advanced Process Technology and Urban Resource Efficiency (CAPTURE), Frieda Saeysstraat, B-9052 Ghent, Belgium

**Keywords:** Self-healing concrete, Fungi, Biomineralization, Calcium carbonate

## Abstract

Concrete is the most used construction material worldwide due to its abundant availability and inherent ease of manufacturing and application. However, the material bears several drawbacks such as the high susceptibility for crack formation, leading to reinforcement corrosion and structural degradation. Extensive research has therefore been performed on the use of microorganisms for biologically mediated self-healing of concrete by means of CaCO_3_ precipitation. Recently, filamentous fungi have been recognized as high-potential microorganisms for this application as their hyphae grow in an interwoven three-dimensional network which serves as nucleation site for CaCO_3_ precipitation to heal the crack. This potential is corroborated by the current state of the art on fungi-mediated self-healing concrete, which is not yet extensive but valuable to direct further research. In this review, we aim to broaden the perspectives on the use of fungi for concrete self-healing applications by first summarizing the major progress made in the field of microbial self-healing of concrete and then discussing pioneering work that has been done with fungi. Starting from insights and hypotheses on the types and principles of biomineralization that occur during microbial self-healing, novel potentially promising candidate species are proposed based on their abilities to promote CaCO_3_ formation or to survive in extreme conditions that are relevant for concrete. Additionally, an overview will be provided on the challenges, knowledge gaps and future perspectives in the field of fungi-mediated self-healing concrete.

## Introduction

Although concrete is one of the most widely used construction materials, used in 80% of the construction cases [1, 2], the related durability issues cannot be neglected. Due to its shrinkage during hardening, its low tensile strength and brittle behaviour, concrete typically suffers from crack formation. These cracks include drying shrinkage cracks (caused by water evaporation), plastic shrinkage cracks (the shrinkage stress exceeds the material’s ultimate tensile strength), thermal cracks (due to temperature variations), load cracks (due to tension, compression, shear or torsion), construction cracks (caused by poor construction quality, for example in pouring or in transportation) and settlement cracks (due to an uneven settlement) [3]. Over time, the entry of water, oxygen and CO_2_ will lead to freeze–thaw damage, chemical attack, reinforcement corrosion and consequently internal expansion caused by corrosion products, all endangering the material’s durability [4, 5]. Reinforcement corrosion is an issue of major concern: once initiated, it progresses and shortens the service life of the structure by causing surface cracking and subsequently spalling of the concrete cover due to expansion of the corroding steel [6]. As crack formation is an inherent flaw of concrete, costs for inspection, maintenance and renovation are inevitably high [5]. Moreover, more than 4 billion tonnes of cement are produced yearly [7], the production of 1 ton of cement releases approximately 0.6 ton of CO_2_ [8], and accounts for 8% of the global anthropogenic CO_2_, caused by the calcination of limestone and fuel combustion [1]. In other words, the construction sector, and more specifically the subsector producing non-durable concrete structures, poses critical challenges at a global scale, in terms of both economic and environmental sustainability.

Concrete is a mixture consisting of fine and coarse aggregates, water and cement with the latter component playing a crucial role as a binder during the concrete hardening. To maximize the lifespan and thus sustainability of concrete structures, deteriorating cracks should be avoided as much as possible. Concrete possesses an inherent ability to heal small cracks autogenously by two major mechanisms: (i) carbonation and (ii) the further hydration of unhydrated cementitious material near the cracks [9, 10]. Carbonation results from the reaction between atmospheric CO_2_ and portlandite (Ca(OH)_2_) (Eq. 1), which is, after calcium silicate hydrates (C–S–H), the major hydration product of concrete. Because of the presence of portlandite, concrete has a very alkaline nature, resulting in high pH values up to 13. C–S–H account for the concrete’s strength.

1 $${\text{CO}}_{2} + {\text{ Ca}}\left( {{\text{OH}}} \right)_{2} \to {\text{CaCO}}_{3} + {\text{ H}}_{2} {\text{O}}$$

Thanks to its efficient bonding capacity and high compatibility with cementitious materials, given its autogenous presence, calcium carbonate (CaCO_3_) is considered as one of the most suitable self-healing products [11]. Calcium carbonate has three anhydrous crystalline polymorphs: calcite, aragonite and vaterite. Calcite is thermodynamically the most stable and most common form [12]. The second mechanism, further hydration, relies on the presence of unhydrated cement particles, which always remain present in a hardened cementitious matrix. As water flows into the cracks, continued hydration of these unhydrated cement grains produces new C–S–H, thereby resulting in crack sealing. Autogenous self-healing can be enhanced by incorporating fibres, adding mineral admixtures, using curing agents or dispersing water-absorbing polymers, however, it never allows the healing of cracks with a width larger than 0.2 mm [4, 9, 13, 14], necessitating the use of self-healing agents, such as microorganisms. Emergent research in biocementation aims at substituting cement with more sustainable microbially induced calcite precipitation (MICP) [15, 16], thereby strengthening the concrete and enhancing the durability of concrete by leading to self-healing of larger-sized cracks [2, 5, 11, 16–23]. The process of biocementation is based on the precipitation of CaCO_3_ on sand grains [24]. Materials are produced by inoculating microbes in sand or soil, which is then repeatedly flushed [25] or immersed [25, 26] with a cementation solution containing calcium ions and urea, causing the microorganism-containing aggregates to solidify [27]. Given that certain fungi and bacteria have a native capability of CaCO_3_-producing biomineralization, they are considered to be efficient strengthening [28] or self-healing [29, 30] agents and this has given rise to the development of applications in which microbial cells and nutrients are added to the concrete mix prior to its use, as a prevention measure. Preferably, spores are added as they are metabolically inactive, highly resistant to adverse conditions typical for the concrete environment and remain viable for prolonged time periods [5, 23]. Crack formation exposes the spores to water and oxygen, thereby inducing spore germination with vegetative growth of the microbial cells giving rise to biomineralization of CaCO_3_ and healing of the crack [5, 23]. This CaCO_3_ precipitation takes place at the microbial cell wall surface that serves as nucleation site [22].

Instead of being applied during the fabrication of concrete, calcite biomineralizing treatments could also be applied to existing concrete structures, for example as a surface treatment, which has much potential in contributing to the protection of cultural heritage building materials from damage and deterioration [31], especially under conditions requiring long-term reliability, for example because of a poor accessibility to the infrastructure, or under conditions requiring regular maintenance [32]. Besides concrete, microbial self-healing could be applied for geological materials such as limestone or sandstone, which are of high interest as well when considering historical buildings, although this has hardly been investigated [31].

**Fig. 1:**
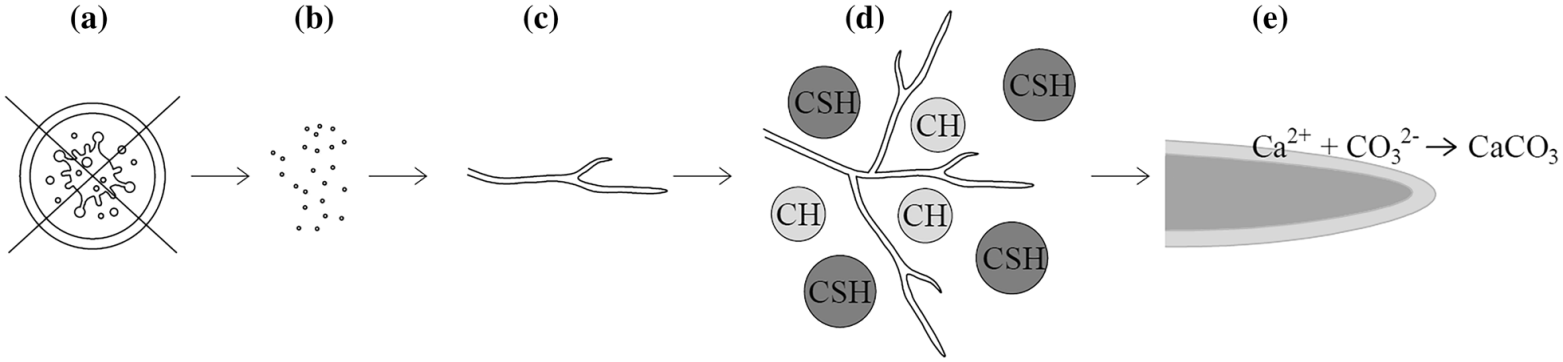
Schematic overview of the selection criteria for microbial strains to be used as self-healing agents in MICP. The microorganism should **a** be non-pathogenic, **b** be sporulating, **c** be capable of vegetative growth (for example, with a filamentous cellular morphology), **d** be able to grow in the alkaline concrete environment (CH: calcium hydroxide, C–S–H: calcium silicate hydrates) and **e** promote CaCO_3_ precipitation (zoom on hyphal tip)

MICP has been demonstrated for various microbial species. While much research has been performed on the use of bacteria as self-healing agents [18, 22, 29, 33, 34], recently the application has been extended towards fungi as well [5, 23, 35]. Upon selecting suitable strains, it should be taken into account that the deleterious alkaline environment of concrete severely complicates microbial survival and growth. The fungi should thus be able to prosper in a high alkaline environment, such as alkaliphilic species do. Therefore, and because of the requirements for the self-healing function, the characteristics of the selected species or strain for use as a self-healing agent in concrete should meet the following stringent criteria: (a) it should be a non-pathogenic strain, (b) it should be a sporulating strain producing highly resistant spores, (c) it should be capable of vegetative growth in conditions relevant for crack formation, (d) it should be capable of surviving and growing in a harsh alkaline environment and (e) it should promote the precipitation of CaCO_3_ at the cell wall surface (Fig. 1).

The concrete structures targeted for applications with fungi-mediated self-healing are the ones subjected to or in contact with water and salts such as chlorides, which can be harmful to the steel reinforcement, and where access for repair or maintenance is limited. Examples of such structures are water-retaining walls, bridges, marine structures, wastewater treatment plants, tunnels and underground parking garages. However, existing structures can be targeted as well, in which case cracks could be healed by means of a repair mortar or spray containing fungal spores (and possibly nutrients), as already done with bacteria. This investigation will already give first insights on CaCO_3_ precipitation and the (positive) influence on the survival and growth of the fungal spores, as they will not be incorporated in the concrete mix.

In this review, we present a comprehensive overview of the state-of-the-art of microbial self-healing of concrete, thereby drawing a conceptual framework, which is required to summarize and further explore the potential of filamentous fungi for this biotechnological application. The review paper starts with the state-of-the-art of bacteria-based self-healing concrete, followed by fungi. From this point onwards, the focus is placed on fungi only. Conceptual principles and hypotheses on fungal CaCO_3_ precipitation in concrete are explained, applications of biomineralization processes in practice are given and a list of potential candidates is identified. Finally, the challenges, knowledge gaps and future perspectives for fungal-based self-healing concrete are discussed.

## State of the art in bacteria-mediated self-healing of concrete

The use of bacteria in concrete self-healing by means of MICP is well-established. Typically, endospore-forming chemoorganotrophic *Bacillus* species are used [14]. Two major different metabolic strategies have been identified to underly the induction of calcite precipitation: the ureolytic and non-ureolytic pathway. The ureolytic pathway requires the enzyme urease to catalyse the hydrolysis of urea (CO(NH_2_)_2_) into ammonium (NH_4_^+^) and carbonate (CO_3_^2−^), which then react with Ca^2+^-ions that are sequestered by the bacteria from the concrete environment and deposited on their negatively charged cell wall surface. The reaction subsequently leads to the precipitation of CaCO_3_ at the cell wall surface.

However, not all bacteria are capable of synthesizing urease and, depending on the application, the growth and survival of many ureolytic bacteria can be inhibited [36]. Furthermore, NH_4_^+^ is known to be an environmental pollutant and poses a health risk for animals and humans [2]. Therefore, the use of non-ureolytic bacteria, which is based on the metabolic conversion of an organic calcium source through bacterial respiration leading to CO_2_ production and calcium carbonate precipitation [29], is a more environment-friendly approach. The addition of an organic calcium source positively influences the concentration of calcium ions and subsequently the amount of self-healing products, thereby increasing the self-healing efficiency. Additionally, depending on the type of source and the amount added, concrete’s mechanical properties are influenced as well [18, 21, 37–40]. The results in literature are however inconsistent, which can be related to different reasons: the method with which the calcium source is added to the concrete mix, the concentration of bacterial spores, the nutrients, the type of cement, etc. Frequently proposed organic nutrients are calcium formate, calcium lactate, calcium glutamate and calcium acetate.

The direct addition of bacterial spores and nutrients to the concrete mix has been shown to result in a decreased intrinsic compressive strength of the material and in a loss of viability of the spores [2]. The reduced cell’s functionality is related to the mechanical forces during the mixing process, a decrease in matrix pore diameter due to cement hydration and to the harsh alkaline environment [2, 21, 41]. Indeed, the typical diameter of bacterial spores used for the self-healing application ranges between 0.8 µm and 1 µm, whereas the pore diameter size in 28 days cured concrete specimens ranges between 0.01 µm and 0.1 µm [21]. Both bacterial spores and nutrients should thus be protected; therefore, several encapsulation techniques have been reported in literature. For example, bacterial spores and calcium lactate could be immobilized in expanded clay particles [29], which served both as a protective barrier and as a structural element of the material. With this technique, a concrete crack width up to 0.46 mm could be healed, whereas healing in the control sample was limited to a crack width of 0.18 mm [29]. In the study of Wang et al*.*, bacterial spores were encapsulated into hydrogels: the bacterial and non-bacterial series resulted in a maximum healed crack width of respectively 0.5 mm and 0.3 mm and in a decrease in water permeability of respectively 68% and 15–55% on average [20]. The use of hydrogels for encapsulation purposes has recently been extended by adding natural polysaccharides such as alginate and cellulose. Given the biodegradable nature of these compounds [2], a sustainable alternative is provided with respect to conventional polymers, while at the same time possibly providing an additional nutrient source for the microbial host.

Besides adding bacterial spores in the concrete mix, existing concrete infrastructures can be targeted as well by applying a spore-enriched repair mortar or repair spray in the crack. In the latter case, the previous stated issues related to the mechanical forces, pore diameter size, alkaline environment and the limited availability of oxygen and nutrients do not apply, thus increasing the chances for an effective self-healing procedure. Van Tittelboom et al*.* used silica gel to immobilize ureolytic bacteria while at the same time filling the cracks before the start of CaCO_3_ crystal precipitation inside the matrix [22]. After placing the concrete samples in an equimolar urea-calcium solution for 3 days followed by a drying period of 3 days, complete filling of the cracks and crack bridging was witnessed for the samples with a crack width of 0.3 mm [22].

The study of bacteria-based self-healing of concrete has gone beyond lab-scale experiments. Successful field trials have been performed for different types of structures and repair techniques [29, 33]. These and other promising results led to the development of the spinoff company Basilisk Self-Healing concrete in the Netherlands, which proposes three different repair techniques: a self-healing admixture for new structures, a self-healing repair mortar and a liquid repair system for existing structures. These technologies currently enable the healing of cracks of up to 1 mm [30].

## State of the art in fungi-mediated self-healing of concrete

Despite successful developments in the use of bacteria for biogenic crack repair in concrete, applications are not yet far-reaching. An extension towards other microbial hosts is the way forward and given the versatile lifestyles and morphologies of filamentous fungi, these appear to be promising candidates to target. Recently, pioneering studies have been performed with different fungal strains to investigate their eligibility for the concrete self-healing application [5, 23, 35]. Various species harbouring different lifestyles were selected for these initial screening experiments. On one hand, well-characterized species such as *Trichoderma reesei* and *Aspergillus nidulans* were investigated [5, 23], for which biological information, including genome sequence data, is available. Indeed, the availability of sufficient biological information about the species under consideration for biotechnological application in concrete self-healing could be considered as an additional criterion next to criteria defined previously (Fig. 1). On the other hand, less well-known species were selected, for example endophytic fungi that live in association with plant roots in nutrient-poor environments such as *Umbelopsis dimorpha* and *Pseudophialophora magnispora* [15]. These selected species belong to different phylogenetic groups within the fungal kingdom [5, 23]. Both *T. reesei* and *A. nidulans* belong to the phylum Ascomycota, which differs from the phylum Basidiomycota in many aspects besides morphological features, as recently discussed from a phylogenetic perspective [42, 43]. For example, the formation of the fruiting body by basidiomycetes does not make them as attractive as ascomycetes for the application. Both phyla are part of the taxonomic group Dikarya, a subkingdom of the fungal kingdom [44, 45].

Initial growth set-ups were made to investigate the compatibility of fungal growth with the concrete environment and in case growth was observed, X-ray diffraction (XRD) and scanning electron microscopy with energy dispersive X-ray spectroscopy (SEM–EDX) were used to determine if calcite precipitation had occurred [4, 15]. XRD is a technique to characterize crystalline materials by providing information about the structures, phases, the preferred crystal orientations, and other structural parameters such as crystallinity [46]. SEM–EDX is used to visualize the surface of fungal precipitates and to characterize its composition and morphology [23]. It was shown that the spores of *T. reesei* germinated on concrete and resulted in the growth of mycelium, which grew equally well with or without concrete [15]. Interestingly, temperature was a crucial factor as growth on concrete was observed at 30 °C but not at 25 °C. Solid precipitates were shown to be associated with the fungal hyphae and were confirmed to be composed of calcite [15]. Similar successful results were obtained for *A. nidulans* [4], however, in this case a genetic engineering approach was used to target the *pacC* gene encoding a pH regulatory transcription factor thereby generating an alkalitolerant phenotype compensating for the alkaline concrete environment [5]. *A. nidulans* is a biosafety level 1 fungus, and its genetic engineering opens new prospects for applications within the context of the circular economy such as concrete self-healing [47]. However, as is the case for any application with genetically modified organisms (GMOs) for which there is a risk of environmental release, it will be necessary to align the development of real-life applications with ethical and regulatory requirements. Another study demonstrated that the strain *Fusarium oxysporum* was also able to grow on a layer of hardened concrete and precipitate CaCO_3_, SEM images and FTIR (Fourier Transform Infrared Spectroscopy) spectra confirmed spore germination and the presence of CaCO_3_ and calcium oxalate monohydrate, resulting from the fungal metabolism [35]. FTIR is a technique used to obtain the infrared spectrum of transmission or absorption of a sample which reveals the sample’s composition [48]. Notably, *T. reesei*, *A. nidulans* and *F. oxysporum* are Biosafety Level 1 (BSL 1) fungi [49].

## Mechanisms of fungal biomineralization relevant for the application of self-healing concrete

**Fig. 2:**
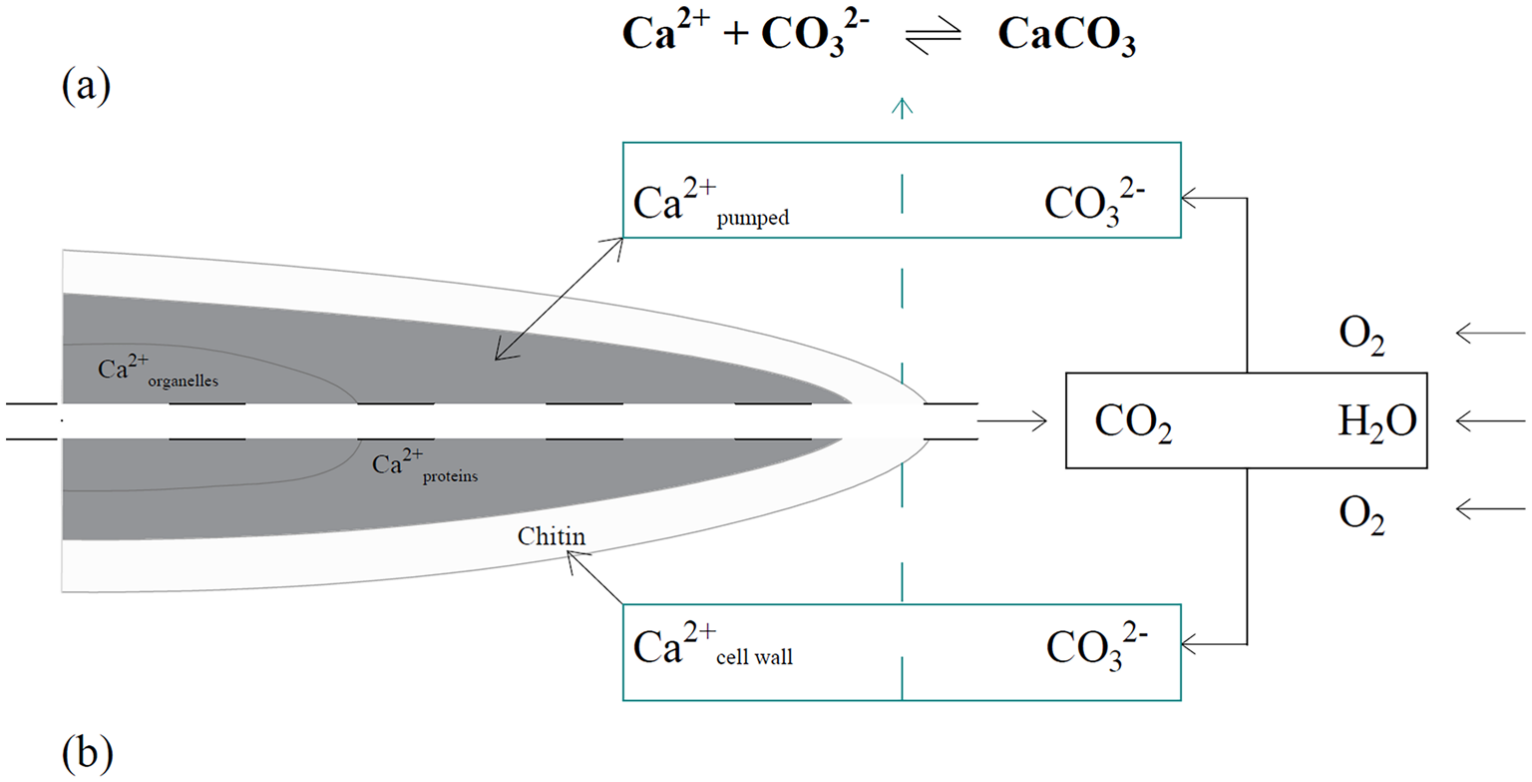
Schematic principles of the biomineralization process in concrete by fungi on microscale illustrated by a zoom on a hyphal tip inside a concrete crack: **a** induced biomineralization and **b** organomineralization. The figure is adapted from (45)

Fungi are involved in the promotion of mineral precipitation of CaCO_3_ through either induced biomineralization (metabolism-dependent) and/or organomineralization (metabolism-independent) processes (Fig. 2). Induced biomineralization of CaCO_3_ is a direct result of the fungal metabolism because it influences the concentration of Ca^2+^-ions and carbonate alkalinity [45] (Fig. 2). Fungi display a tight homeostatic control of the cytoplasmic Ca^2+^ concentration as it regulates the apical growth of the hyphae and participates in several vital intracellular processes such as development and proliferation, stress response and integrity of the cell wall [50, 51]. Calcium homeostasis by fungi occurs mainly in three ways: (i) the active and passive transport of Ca^2+^-ions in/out of the cell (Ca^2+^_pumped_), (ii) the sequestration of cytoplasmic Ca^2+^ into specific organelles (Ca^2+^_organelles_) and (iii) binding to calmodulins (CaM) and calcineurins (Ca^2+^_proteins_) [50]. The pumping is performed by specific channels and antiporters fine-tune the intracellular calcium concentration [51]. Ca^2+^-ATPase are transporters capable of sequestering calcium and directing it to different organelles, such as vacuoles, Golgi and ER, decreasing cytosolic calcium levels [51]. Calmodulins are cytoplasmic proteins that change their structural conformation in response to intracellular Ca^2+^ levels, forming a Ca^2+^/CaM complex. This complex becomes the binding target for several other proteins that activate specific kinases and initiate signalling cascades [52]. Calcineurins are phosphatases that activate specific transcription factors and activate signalling cascades that are involved in various biological processes [51]. On the other hand, fungal metabolic activities can increase carbonate alkalinity because of water consumption, physicochemical degassing of fungal respired CO_2_, oxidation of organic acids, nitrate assimilation or urea mineralization [45]. Overall, the extent of MICP is thus dependent on (i) the extracellular calcium concentration, which in turn is dependent on the homeostatic control of the fungus, (ii) the concentration of extracellular carbonate ions and (iii) the availability of nucleation sites [53–55]. Importantly, the concentration of carbonates present in the extracellular medium is dependent on the alkaline pH of the external environment and dissolved inorganic carbon (DIC), which in turn depends on the metabolic activities of the fungus, as previously described. It should also be taken into account that high intracellular Ca^2+^ concentrations may result in cytotoxicity [45]. Fungi may therefore protect themselves by the extracellular precipitation of CaCO_3_ to decrease the excess of intracellular Ca^2+^ concentration [45] and thus by making the calcium insoluble.

Fungi-mediated CaCO_3_ precipitation through organomineralization relies on their metal-uptake or biosorption capability thanks to the presence of chitin, a long-chain N-acetylglucosamine polymer, in their cell walls, which confers structural rigidity to the cell [56]. Chitin forms a substrate on which calcite may nucleate and subsequently grow, thus functioning as a catalyser to promote crystal nucleation [56–59] (Fig. 2). The polymer is known for its ability to bind Ca^2+^-ions (Ca^2+^_cell wall_), thus reducing the activation energies required for nuclei formation [5, 45]. These bound Ca^2+^-ions will then react with CO_3_^2−^-ions resulting in the precipitation of CaCO_3_ on the hyphae. This process is metabolism-independent and could thus occur for both metabolically active and inactive fungi [5, 23, 45].

Fungal excretion of organic acids, more precisely oxalic acid, is another form of organomineralization leading to CaCO_3_ precipitation. Oxalic acid, a simple organic di-acid, is a by-product from the fungal metabolism like the glycoxylate cycle [60]. More commonly found in its oxalate salt form, this compound is toxic for the living cells, thus, needs to be expelled by specific transporters or be degraded [60]. When secreted, oxalates have a remarkable ability to complex with metals. Thus, in the presence of rock and mineral-based substrates, fungal secretion of oxalic acid can induce metal oxalate precipitation, with calcium oxalate being the most ubiquitous [60, 61]. This microbe-mediated mineral dissolution of the limestone matrix's internal pore walls and other limestone-like rocks favours the increase in carbonate concentration [23, 62]. Finally, also ureolytic fungi have been described [54, 63].

## Applications based on fungal biomineralization

Biomineralization such as CaCO_3_ precipitation and the ability to take up metals by fungi has already been explored for other applications. Xie et al*.* reported the role of fungi in the development of the naturally formed Rimstone Dams in the Huanglong park area of the Sichuan Province of China [64]. Fungal hyphae serve as nucleation points for the formation of crystalline CaCO_3_, which then grows to fuse into calcite plates resulting in the main structural framework of the travertine dams in this area [64]. Crystalline structural analysis of the travertine proved its biological origin and the crystals each showed a hole in their core where a fungal hyphae used to reside [64]. From the retrieved samples, examination showed that the oomycetes *Pythium* and *Saprolegnia* were dominantly present, with oomycetes being a group of filamentous fungal-like eukaryotes that are phylogenetically more closely related to algae [65]. Fungal species found included *Phoma* sp., *Mucor* sp. and *Botrytis* sp.

On the other hand, fungal biosorption could be employed for the removal of heavy metals, such as cadmium, chromium, copper, lead, mercury, nickel, palladium and zinc from wastewater and the environment, thereby providing a cheaper and environment-friendly alternative approach to the conventional treatments using physicochemical techniques. Indeed, the large surface area of fungal cells and the presence of negatively charged structures in their cell wall offer multiple metal-binding active sites to bind metal cations [66, 67].

Many species are also able to thrive in environments that are highly polluted by metals, such as the fungi used as a biosorbent for heavy metals removal from wastewater [5, 66].

## Hypotheses underlying a high potential of fungi for self-healing of concrete

Based on the initial observations with respect to the use of *T. reesei*, *A. nidulans and F. oxysporum* for concrete self-healing [5, 23, 35], and the more extensive knowledge on bacteria-based self-healing concrete [2, 11, 16–22], conceptual principles and hypotheses can be drawn for the use of filamentous fungi in these applications. Overall, these microorganisms appear to have a large potential for such applications. Hyphal growth into a three-dimensional network could facilitate the fast colonization of cracks and high rates of calcite precipitation. Furthermore, fungi are already known for their capability of calcite precipitation, not only through biomineralization, but also by means of organomineralization [45].

**Fig. 3:**
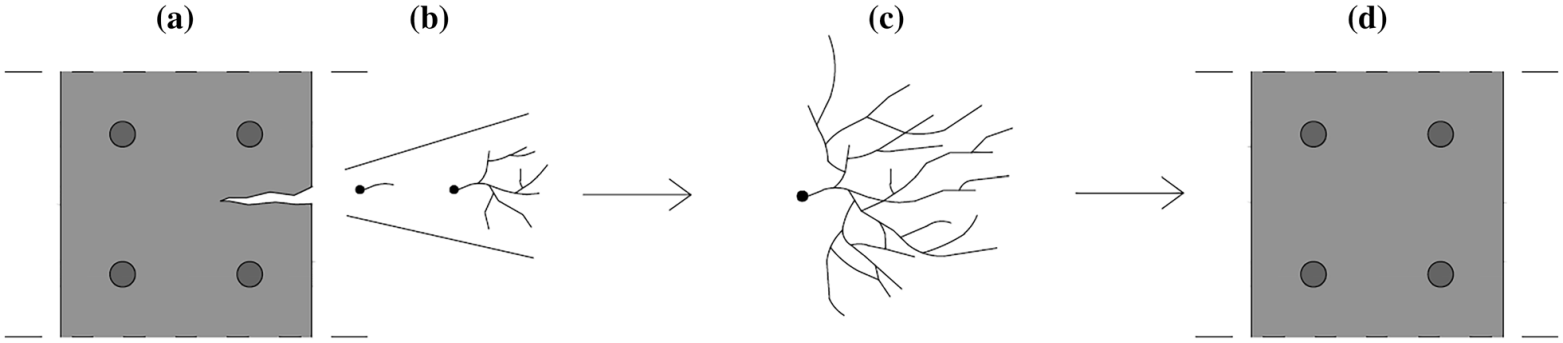
Schematic overview of the conceptual principles on macroscale for fungi-mediated self-healing concrete: **a** reinforced concrete containing fungal spores with a crack, **b** germinating spores, **c** growing mycelium with CaCO_3_ precipitation on hyphae and **e** self-healed concrete

The concept of fungal-based CaCO_3_ precipitation in concrete can be explained as follows (Fig. 3). First, fungal spores and nutrients are added to the concrete mix, either directly or through encapsulation, where the spores represent an inactive dormant state of the organism. Next, upon crack formation, ingress of water and oxygen will reactivate the fungal metabolism, inducing their germination into fungal hyphae. These hyphae grow in extensive thread-like three-dimensional mycelium networks. Fungal mycelia can cover huge areas [68], for example by decomposing wood and dead plant matter resulting in the formation of large networks on the forest floor [69]. These high biomass yields and growth rates are advantages that potentially lead to an increased amount of CaCO_3_ precipitation, also because more nucleation sites are present given the high surface-to-volume ratio of the hyphae. This hypothesis was also put forward by Zhang et al. [35]. Consequently, the self-healing of larger cracks (with a width larger than 1 mm), which cannot be healed with bacteria-mediated processes, can be targeted. Additionally, the presence of hydrophobins in fungal cell walls [70, 71] might provide a water-repellent barrier which is favourable to the concrete. Calcium carbonate precipitation will take place on these hyphae, with the concrete environment providing the required Ca^2+^-ions and the CO_3_^2−^-ions being present either due to the incoming water or due to fungal respiration, resulting in CO_3_^2−^-ions after the dissolution of CO_2_. Finally, the precipitated CaCO_3_ will seal the crack and thus protect the reinforcement from corrosion and the concrete from further damage.

Even though that the intrinsic capability of fungi to perform biomineralization suggests that their implementation for concrete self-healing could be promising, the concrete environment remains detrimental and harsh for microorganisms to grow in due to the high pH value, small-sized pores and lack of nutrients [5]. Fungi are however known for their ability to survive and adapt to extreme environments with for example limited nutrient availability, extreme temperatures, intense ultraviolet light and variable acidity [23, 72, 73]. On the other hand, potential detrimental effects of the acidification of the concrete by the fungi should be discussed as well. When colonising mineral particles, both bacteria and fungi may induce local acidification of the mineral surface ([74, 75]). The high alkalinity of the concrete material is however required to protect the steel reinforcement from corrosion (passivation) and so degradation of the material. Carbonation is for example a significant contributor to concrete deterioration by reducing the pH to a value of 8.5–10 [76]. To minimize the impact of the fungi on the concrete alkalinity, different approaches for implementing the healing mix (fungal spores and nutrients) in the concrete could be considered. The healing mix could be added directly or through encapsulation, or in a repair mix/solution sprayed into the crack. Direct addition of the healing mix could have a negative impact on the pH of concrete, however based on observations that encapsulation is needed for bacteria-mediated concrete self-healing applications, this might not be the preferred method. By encapsulating the healing mix, the spores and nutrients are protected and the mechanical properties of concrete are less affected. Additionally, a pH buffer could be added to maintain the medium at a constant pH. If the healing mix is sprayed into a surface crack, the concrete pH will probably even be less affected as also carbonation will take place starting at the surface. However, research observations are currently lacking for fungi-mediated self-healing concrete. While hypotheses can be made based on the literature on the use of bacteria, investigations on this matter are still required to draw conclusions for fungi.

## Potential candidate fungi for concrete self-healing applications

Fungi are a very species-rich group of eukaryotic organisms with a diversity of at least 1.5 million species, and probably even 3 million [23, 77]. This wide diversity allows us to screen various fungal species as potential eligible candidates for fungi-mediated self-healing concrete (Table 1). Fungal species with lifestyles that are compatible with the concrete environment could include alkaliphilic, ureolytic and/or oligotrophic species.

**Table 1:** Overview of eligible candidates for fungi-mediated self-healing concrete

Fungal species	Interesting characteristics	Source
Aspergillus nidulans*, Trichoderma reesei, Fusarium oxysporum *pH regulatory mutant	Grows on concrete layer CaCO_3_ precipitation	[5, 23, 35]
*Aspergillus sp., Aureobasidium sp., Cephalosporium sp., Fusarium sp., Monilla sp.*	Grows on limestone rock Epi- and endolisthic fungi	[72]
*Cephalotrichum oligotriphicum, Chrysosporium guizhouense, Clonostachys rosea*	Grows in limestone cave Oligotrophic fungi	[81]
*Mortierella sp.*	Grows on moonmilk Oligotrophic fungi	[51]
*Paecilomyces inflatus, Plectosphaerella cucumerina*	Grows on calthemite straw stalactite growing from a concrete ceiling Oligotrophic fungi	[83]
*Chrysosporium sp., Paecillomyces lilacimus*	Grows on media with a maximum pH of 11 Alkaliphilic fungi	[23]
*Neurospora crassa, Penicillium chrysogenum CS1*	CaCO_3_ precipitation in the presence of urea	[54, 63]

A special focus can be placed on alkaliphiles, which grow well at pH values higher than pH 9, with optimal growth typically observed at a pH value between 10 and 13, whereas slow or no growth is observed at near-neutral pH values [78, 79]. Alkaliphilic fungi are found in soda soils, characterized by pH values higher than 8 due to the presence of sodium carbonate (Na_2_CO_3_), thereby representing an extreme habitat in which growth of most other organisms is restricted [78, 80]. Examples of alkaliphilic fungi are *Paecilomyces lilacimus* and *Chrysosporium spp.*, which are able to grow at a maximum pH value of 11 [23].

Another criterium for species selection is the inherent metabolic capability of performing MICP, such as the presence of urease. For example, it was demonstrated that the ureolytic fungus *Neurospora crassa* mediates the formation of calcite in urea- and calcium-containing media [54]: here, more than 90% of the calcium present in the media was removed by the fungal biomass and could react with dissolved carbonate. In another example, the ureolytic fungi *Penicillium chrysogenum CS1* was isolated from cement sludge and shown to promote the formation of biosandstone by calcite precipitation [63]. For the use of urease-positive fungi in self-healing concrete urea should thus be added to the healing mix which contains the fungal spores and possibly a calcium source and nutrients.

Based on the poor nutritional status of the concrete environment, oligotrophic fungi could be selected. For example, oligotrophic fungi can be found in natural habitats such as caves, characterized by constant low temperature, high humidity, lack of organic matter and darkness [81]. This type of fungi thus has a high potential to prosper in a concrete environment. Starting from air, limestone, water and soil samples from a carbonate cave, the species *Plectosphaerella cucumerina*, *Clonostachys rosea, Cephalotrichum oligotriphicum* and *C. guizhouense* were isolated [81]. Interestingly, these species were able to grow on a carbon-free medium.

Park et al*.* investigated the microbial diversity of moonmilk, a type of speleothem found in limestone caves. Speleothems are secondary deposits in caves and are composed of one or a mixture of the minerals calcite (CaCO_3_), aragonite (CaCO_3_) and gypsum (CaSO_4_·2H_2_O) with calcite the most prominent [82]. As recent studies suggest a strong potential for eukaryotic activities to influence moonmilk formation [51], the authors indeed found the fungus *Mortierella* to be dominantly present in moonmilk.

Another oligotrophic environment are rock substrates due to the scarcity of nutrients and moisture and their exposure to solar radiation [72]. Epi- and endolithic fungi are able to colonise rock surfaces and pre-existing cracks and fissures, constituting a significant part of the microflora in a wide range of rocks. Known fungal genera found in limestone rock substrates are *Aspergillus*, *Aureobasidium*, *Cephalosporium*, *Fusarium*, *Monilla* and *Penicillium* [72].

Finally, anthropogenic habitats could be explored for the presence of fungal species with potential for concrete self-healing. Pasquale et al*.* isolated two ascomycota fungi from a calthemite straw stalactite growing from a concrete ceiling of a building, *Paecilomyces inflatus* and *Plectosphaerella cucumerina* [83]. After laboratory incubation, SEM and XRD analyses showed that the crystals precipitated on the fungal hyphae were vaterite and calcite in the presence of *P. inflatus* and pure calcite in the presence of *P. cucumerina* [83].

## Future perspectives and potential applications

Despite the promising initial proof of concept studies demonstrating growth and CaCO_3_ formation with the fungi *T. reesei*, *A. nidulans* and *F. oxysporum*, the current state-of-the-art is still lacking in-depth and more elaborated research. The methods by which fungal spores can be implemented in the concrete mix remain unexplored; up till now, no literature exists on the addition of fungal spores during the mixing process of concrete. Both direct addition and encapsulation methods can be considered, each giving rise to new research questions. Can fungal spores survive the harsh concrete environment? How should the nutrients be added? Which concentration of fungal spores (and nutrients) can be added without compromising concrete’s mechanical properties? In case of encapsulation, which techniques can be applied to fungi? Can the protective barrier simultaneously act as a nutrient? Additionally, concrete can have many different compositions, each of them affecting the spores’ survivability and growth capabilities in a different way, thus imposing an additional challenge. As a result of this knowledge gap, the organism’s influence on the mechanical, physical and chemical properties of concrete has not yet been investigated, neither the organism’s growth capabilities when surrounded by a concrete environment. Consequently, no research exists on the crack width that could possibly be healed with the help of fungi. However, thanks to their high biomass yield and growth rates, it is aimed to heal cracks with a width larger than 1 mm. Nevertheless, the potential of various eligible fungal candidates remains undetermined and currently only three fungal species have shown favourable results. The fungal growth rate and the associated CaCO_3_ precipitation rate in concrete remain unknown as well, although they play a crucial role in the protection of the concrete material against the entry of water.

Future perspectives should thus include research and development approaches that are capable of tackling these challenges and knowledge gaps. Based on the research on bacteria-based self-healing concrete, several encapsulation techniques can be tested through an experimental investigation on fungal spores to assess their survivability, growth and CaCO_3_ precipitation upon crack formation. Results can be compared with direct addition of the spores. This kind of research will allow the investigation of other knowledge gaps, such as the maximum crack width that can be healed and the efficacy of the precipitated CaCO_3_. Furthermore, research on bacteria-fungi interactions for CaCO_3_ precipitation is inexistent but could potentially mediate challenges encountered with both bacteria and fungi. In nature, bacteria and fungi co-inhabit in harsh and stressful environments [84, 85]. Research showed the stimulation of bacterial activity by mycelial supply of scarce resources in dry and nutrient-free environments [86]. Indeed, fungi possess higher resistance to drought and nutrient limitation [87, 88], possibly also in concrete, and mycelia can redistribute water and nutrients due to its efficient resource translocation [89]. Moreover, some bacterial strains are known to hitchhike along fungal highways [90]. The strategy is already used to mobilize pollutant-degrading bacteria around fungal hyphae in polluted soil [91]. This principle deserves further research in the context of self-healing materials because possibly fungi could serve as vectors for the dispersion of biomineralizing bacteria and nutrients in cracks. Additionally, no basidiomycete fungi able to promote the precipitation of CaCO_3_ has been described in the literature (Table 1), although some of these species are known to live in environments with high levels of oxidative stress. The investigation on basidiomycetes in the context of CaCO_3_ precipitation could therefore also be an interesting point of research.

## Conclusion

This review provides conceptual principles and insights into the potential of fungi for self-healing of concrete cracks with a focus on fungal biomineralization processes, while providing an overview of potential fungal candidates suitable for the application. In contrast to bacteria, filamentous fungi grow in extensive mycelium networks with branched filamentous-shaped hyphal structures that enable to fill and deposit CaCO_3_ in larger cracks. We propose that fungi have a superior ability for concrete self-healing as compared to bacteria, for the following reasons: (i) their capability of forming extensive network-like structures, (ii) their high biomass yield providing abundant nucleation sites for CaCO_3_ precipitation both directly and indirectly, (iii) their nutritional versatility, (iv) their capability of growing in harsh environments relevant for concrete, (v) superior wall-binding and metal-uptake ability because of the presence of chitin in their cell walls, and (vi) the inherent hydrophobicity of fungal cells.

## Data Availability

Not applicable.
